# Genetic characterization of 12 heterologous microsatellite markers for the giant tropical tree *Cariniana legalis* (Lecythidaceae)

**DOI:** 10.1590/S1415-47572010000100022

**Published:** 2010-03-01

**Authors:** Marcela Corbo Guidugli, Klaus Alvaro Guerrieri Accoroni, Moacyr Antonio Mestriner, Eucleia Primo Betioli Contel, Carlos Alberto Martinez, Ana Lilia Alzate-Marin

**Affiliations:** 1 Departamento de Genética, Faculdade de Medicina de Ribeirão Preto, Universidade de São Paulo, Ribeirão Preto, SPBrazil; 2 Departamento de Biologia, Faculdade de Filosofia Ciências e Letras de Ribeirão Preto, Universidade de São Paulo, Ribeirão Preto, SPBrazil

**Keywords:** conservation, jequitibá rosa, population genetics, SSR markers, transferability

## Abstract

Twelve microsatellite loci previously developed in the tropical tree *Cariniana estrellensis* were genetically characterized in *Cariniana legalis*. Polymorphisms were assessed in 28 *C. legalis* individuals found between the Pardo and Mogi-Guaçu River basins in the state of São Paulo, Brazil. Of the 12 loci, 10 were polymorphic and exhibited Mendelian inheritance. The allelic richness at each locus ranged from 2-11, with an average of 7 alleles per locus, and the expected heterozygosity ranged from 0.07-0.88. These loci showed a high probability of paternity exclusion. The characteristics of these heterologous microsatellite markers indicate that they are suitable tools for investigating questions concerning population genetics in *C*. *legalis*.

*Cariniana legalis* (Mart.) Kuntze, commonly known as jequitibá rosa, is a woody tree species of the Lecythidaceae family with late successional characteristics (Lorenzi, 2002). Its flowers are hermaphroditic and are pollinated by bees; dispersion is anemochorous (Sebbenn *et al.*, 2000). Jequitibá rosa is known as one of the giant trees of the Atlantic Rain Forest, reaching 60 m in height and 4 m in diameter (Kageyama *et al.*, 2003). In Brazil, *C. legalis* wood is widely used for general construction purposes and carpentry (Lorenzi, 2002). Unfortunately, the *C. legalis* population has been dramatically reduced by habitat destruction and exploitation of its timber (Sebbenn *et al.*, 2000). To develop strategies for the sustainable management and conservation of this species, an understanding of the effects of spatial isolation on genetic diversity and gene flow is crucial. Although allozymes have been surveyed in *C. legalis* for purposes such as assessing its mating system (Sebbenn *et al.*, 2000), very little is known about the fine-scale population structure of this species.

Simple sequence repeat (SSR) markers have become the marker class of choice for population genetic studies because they are (mostly) co-dominant, abundant in genomes, highly reproducible, and some have high rates of transferability across species (Saha *et al.*, 2006). The widespread use of microsatellites has been limited by the fact that the initial identification of the marker is expensive. Consequently, researchers have tried to use microsatellite markers developed for one species in other species by means of transferability analysis (Roa *et al.*, 2000). Recently, Guidugli *et al.* (2008) developed 15 polymorphic microsatellite markers for *Cariniana estrellensis* and demonstrated the transferability of 12 of these primer pairs to *C. legalis* based on amplification of just seven individuals. Therefore, in this study, characteristics of 12 heterologous loci were investigated using 28 *C. legalis* individuals in order to confirm the general applicability of these markers for genetic studies of this species.

For the genetic analysis, we collected leaves from 28 reproductive *C. legalis* adults located between the Pardo and Mogi-Guaçu River basins in the state of São Paulo in southeastern Brazil. This large area, the Ribeirão Preto region, is highly impacted by agricultural practices (Figure 1). The collection of leaves from the tall trees was a very laborious task, requiring the employment of specialized tree climbers. The geographic coordinates of all sampled individuals were recorded using GPS (GARMIN eTrex^®^ Vista Cx), and the plant tissues were stored at -20 °C until DNA extraction. Total genomic DNA was extracted from frozen leaf tissues based on the protocol described by Doyle and Doyle (1990), with minor modifications. Purity and concentration of the genomic DNA were determined with a spectrophotometer (Spectronic Genesys 5).

Each primer pair previously transferred to *C. legalis* from *C. estrellensis* (Guidugli *et al.*, 2008) was screened with polymerase chain reactions (PCR) at 13 annealing temperatures (between 46-58 °C) in 10 *C. legalis* individuals in order to optimize heterologous amplification. PCR amplifications were carried out in a final reaction volume of 10 μL containing 0.3 μM of each primer, 1 U *Taq* DNA polymerase, 0.25 mM of each dNTP, 1 x MgCl_2_-free reaction buffer [75 mM Tris-HCl pH 9.0, 50 mM KCl and 20 mM (NH_4_)_2_SO_4_], 1.5 mM MgCl_2_ and 2.5 ng of template DNA. Amplifications were performed in a MasterCycler (Eppendorf) according to the following protocol: 5 min at 96 °C followed by 30 cycles of 30 s at 94 °C; 1 min at the annealing temperature defined for each primer (Table 1); 1 min at 72 °C; and a final elongation step of 7 min at 72 °C. PCR products were denatured and separated on 10% denaturing polyacrylamide gels stained with silver nitrate. Allele sizes were estimated using a DNA ladder (10 bp - Invitrogen) and the original DNA clone from which the SSR locus was developed.

Mendelian inheritance was investigated for each locus based on analysis of one mother tree and 20 open-pollinated offspring. Polymorphic loci were characterized with regard to the number of alleles per locus (*A*), as well as expected (*H*_E_) and observed (*H*_O_) heterozygosities for each locus and averaged over all loci, using the GDA software (Lewis and Zaykin, 2002). Hardy-Weinberg equilibrium was tested by Fisher's exact test. FSTAT software package version 2.9.3.2 (Goudet, 2002) was used to test linkage disequilibrium for all loci, applying the Bonferroni correction for multiple comparisons (Rice, 1989). Probabilities of paternity exclusion were estimated using CERVUS version 3.0 (Kalinowski *et al.*, 2007). We also checked the presence of null alleles using the MICRO-CHECKER 2.2.3 program (van Oosterhout *et al.*, 2004).

Our results confirmed the amplification efficiency of the 12 heterologous microsatellite markers for *C. legalis.* Of these, two (16.7%) were monomorphic in the sample of 28 individuals, and the other 10 were polymorphic, showing clear allele size variation (Table 1). Satisfactory amplification products were obtained using eight annealing temperatures ranging from 46-57 °C (Table 1). Mendelian inheritance was verified for all 10 SSR loci in the open-pollinated family. All siblings displayed at least one of the maternal alleles, confirming Mendelian inheritance.

A total of 70 alleles were found at the ten polymorphic microsatellite loci in the 28 reproductive adults. The number of alleles detected in this species ranged from 2 (Ces02) to 11 (Ces03) (Table 1). Most of the successful loci exhibited less allelic variation than was found in the species from which they were developed (average = 8.5; Guidugli *et al.*, 2008). The patterns described above are consistent with earlier studies showing reduced polymorphism in target species as compared to source species in cross-species amplification studies (Ciampi *et al.*, 2008; Feres *et al.*, 2009). According to Peakall *et al.* (1998), related products amplified in different species might include mutations, rearrangements and duplications in the flanking region, causing changes in the number of repeats, which could cause the reduction in polymorphism.

The average observed heterozygosity was 0.32, and expected heterozygosities ranged from 0.07 to 0.88 across loci, with an average of 0.64 (Table 1). This value for average expected heterozygosity in *C. legalis* is higher than that in other tropical trees: *Carapa guianensis* (*H*_E_ = 0.61, Dayanandan *et al.*, 1999), *Swietenia humilis* (*H*_E_ = 0.53, White *et al.*, 1999) and *Solanum lycocarpum* (*H*_E_ = 0.33, Martins *et al.*, 2006). A heterozygote deficiency was observed for the majority of loci, most likely due to the sampling of individuals used for locus characterization (the Wahlund effect) or the presence of null alleles. In fact, the null allele test (MICRO-CHECKER, van Oosterhout *et al.*, 2004) revealed that several loci (Ces02, Ces03, Ces05, Ces12, Ces13, Ces16 and Ces18) showed evidence of null alleles. Furthermore, because the results were obtained using microsatellites developed in a different species, the probability of occurrence of a null allele is much higher than in the case of testing the species from which they were isolated (Kim *et al.*, 2004).

For all loci except Ces14, the genotypic frequencies showed significant departures from Hardy-Weinberg equilibrium (HWE). None of the loci showed significant linkage disequilibrium after a sequential Bonferroni correction for multiple tests, indicating that these markers are appropriate for population genetics studies. The SSR profiles generated by the ten heterologous primer pairs were able to distinguish all 28 *C. legalis* individuals sampled in southeastern Brazil. The first estimate of paternity exclusion probability *Pr*(*Ex*1), when the offspring was sampled but the mother was not, was 0.872 for the combined loci. The second estimate, *Pr*(*Ex*2), when both the mother and the offspring were sampled, was 0.999 over all 10 loci (Table 1). The combined probabilities of paternity exclusion were very high, demonstrating that these markers represent an important tool that will permit more refined and challenging questions regarding gene flow and parentage in natural populations of *C. legalis* to be addressed.

In conclusion, this study characterized a novel set of heterologous microsatellite markers for *C. legalis*. These markers provide a new approach for generating fundamental data important for devising sound conservation procedures for this species.

**Figure 1:**
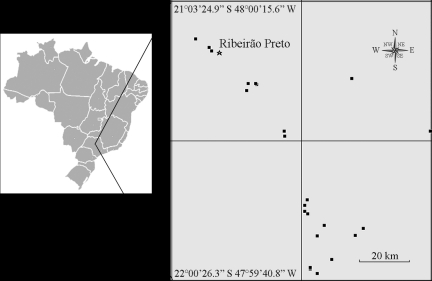
Geographic location of the *Cariniana legalis* individuals sampled in southeastern Brazil for assessment by SSR loci.

## Figures and Tables

**Table 1:** Characteristics of 12 heterologous microsatellite markers for *Cariniana legalis*.

Primer name	Accession number	Primer sequence 5'-3'	Observed fragment size range (bp)	*T*a (°C)	*A*	*H*_O_	*H*_E_	*Pr*(*Ex*1)	*Pr*(*Ex*2)	Dev. HWE
Ces02	EU735166	F:GGACATGGGTTGTTATCCG R:TCTCAACCTCAAAATGTAATAGC	163-165	57	2	0	0.07	0.998	0.967	0.021
Ces03	EU735167	F:GGTGTATCCTAAGGTAGAGC R:CCTTTGGAAGATATGGAAG	207-235	50	11	0.50	0.86	0.460	0.297	< 0.001
Ces05	EU735169	F:CCAGATTGATAAGCTACTCC R:CATTCAAGCAAGTCAAACC	149-163	53	6	0.43	0.73	0.680	0.501	< 0.001
Ces06	EU735170	F:GGGGCATATGTTTATTATTC R:GTGGTAAATCACAAATGTGC	159	51	1	-	-	-	-	-
Ces07	EU735171	F:TTGTAAAAACGGCATGTCC R:GTTCGGAACAGACAAAGAGG	178-202	51	10	0.74	0.87	0.451	0.289	0.009
Ces09	EU735172	F:CAGAGTTTTTCAATAGCGG R:TTTCATTGCAATCATAGGC	185	48	1	-	-	-	-	-
Ces10	EU735173	F:GGGCAGACCAAATCAAGAG R:GCCATTCATAAACCATTCAAG	196-228	49	6	0.44	0.59	0.799	0.621	0.013
Ces12	EU735175	F:ACGCACTTTCTCAATTCC R:TGTAGACTTGTAGGATAAATGG	265-287	46	10	0.26	0.88	0.417	0.261	< 0.001
Ces13	EU735176	F:CTGAAGGGACTGAGGGG R:ATATCAGGAGGTTAAGGGC	142-156	47	5	0.16	0.59	0.817	0.680	< 0.001
Ces14	EU735177	F:CTGGTAAGCTCTTGGTTGTG R:CCATGGGTTTTCTGTTTCC	180-186	51	4	0.24	0.29	0.958	0.846	0.254
Ces16	EU735178	F:GGACATACCTGCCAAAAC R:AGAGTTAGTTGCTGTTATATGG	204-214	51	6	0.07	0.71	0.695	0.514	< 0.001
Ces18	EU735179	F:ATAATCATGACCTGTGCC R:GTCCCTGATCAAGTATGC	166-186	51	10	0.40	0.84	0.499	0.330	< 0.001
Average^1^/Cumulative probability of exclusion^2^						0.32^1^	0.64^1^	0.872^2^	0.999^2^	

*T*a °C, annealing temperature; *A*, total number of alleles per locus; *H*_O_, observed heterozygosity; *H*_E_, expected heterozygosity; *Pr*(*Ex*1) and *Pr*(*Ex*2), paternity exclusion probabilities; HWE, Hardy-Weinberg equilibrium; Significance (p < 0.05). Dev. HWE: deviation from HWE.
